# Cognitive Improvement after Mild Traumatic Brain Injury Measured with Functional Neuroimaging during the Acute Period

**DOI:** 10.1371/journal.pone.0126110

**Published:** 2015-05-11

**Authors:** Glenn R. Wylie, Kalev Freeman, Alex Thomas, Marina Shpaner, Michael OKeefe, Richard Watts, Magdalena R. Naylor

**Affiliations:** 1 Rocco Ortenzio Neuroimaging Center, Kessler Foundation, West Orange, NJ, United States of America; 2 Department of Physical Medicine and Rehabilitation, Rutgers University Medical School, Newark, NJ, United States of America; 3 War Related Illness and Injury Study Center, Department of Veterans’ Affairs, East Orange, NJ, United States of America; 4 Department of Psychiatry, University of Vermont, Burlington, VT, United States of America; University of California, San Francisco, UNITED STATES

## Abstract

Functional neuroimaging studies in mild traumatic brain injury (mTBI) have been largely limited to patients with persistent post-concussive symptoms, utilizing images obtained months to years after the actual head trauma. We sought to distinguish acute and delayed effects of mild traumatic brain injury on working memory functional brain activation patterns < 72 hours after mild traumatic brain injury (mTBI) and again one-week later. We hypothesized that clinical and fMRI measures of working memory would be abnormal in symptomatic mTBI patients assessed < 72 hours after injury, with most patients showing clinical recovery (i.e., improvement in these measures) within 1 week after the initial assessment. We also hypothesized that increased memory workload at 1 week following injury would expose different cortical activation patterns in mTBI patients with persistent post-concussive symptoms, compared to those with full clinical recovery. We performed a prospective, cohort study of working memory in emergency department patients with isolated head injury and clinical diagnosis of concussion, compared to control subjects (both uninjured volunteers and emergency department patients with extremity injuries and no head trauma). The primary outcome of cognitive recovery was defined as resolution of reported cognitive impairment and quantified by scoring the subject’s reported cognitive post-concussive symptoms at 1 week. Secondary outcomes included additional post-concussive symptoms and neurocognitive testing results. We enrolled 46 subjects: 27 with mild TBI and 19 controls. The time of initial neuroimaging was 48 (+22 S.D.) hours after injury (time 1). At follow up (8.7, + 1.2 S.D., days after injury, time 2), 18 of mTBI subjects (64%) reported moderate to complete cognitive recovery, 8 of whom fully recovered between initial and follow-up imaging. fMRI changes from time 1 to time 2 showed an increase in posterior cingulate activation in the mTBI subjects compared to controls. Increases in activation were greater in those mTBI subjects without cognitive recovery. As workload increased in mTBI subjects, activation increased in cortical regions in the right hemisphere. In summary, we found neuroimaging evidence for working memory deficits during the first week following mild traumatic brain injury. Subjects with persistent cognitive symptoms after mTBI had increased requirement for posterior cingulate activation to complete memory tasks at 1 week following a brain injury. These results provide insight into functional activation patterns during initial recovery from mTBI and expose the regional activation networks that may be involved in working memory deficits.

## Introduction

Traumatic Brain Injury (TBI) is a significant medical problem worldwide (e.g., [1]), with as many as 1.7 million people in the US sustaining a head injury each year [2]. Visits to emergency departments in the United States for TBI increased by nearly 30% between 2006–2010 [3]; the surge in visits may be due in part to increasing public awareness, but still represents an important medical problem. The majority (75%) of TBIs are mild (mTBI) [2], and not all individuals who sustain a head injury present to the emergency department. While it appears most individuals with mTBI will recover without lengthy post-concussive syndrome, there are currently no known clinical measurements that can predict which patient will experience persistent deleterious outcomes including cognitive (e.g., attention, executive function, memory), emotional (e.g., depression, anxiety, irritability), somatic (e.g., headache, fatigue, dizziness, pain) and physical deficits [4].

There is a growing literature on the impact of mTBI on the brain using neuroimaging tools [5–17]. Much has been learned through the investigation of changes in the blood oxygen level dependent (BOLD) signal during task performance as well the investigation of changes in functional brain connectivity using the covariation in the BOLD signal from disparate brain regions. Much of the research into task-based brain activation has focused on working memory (WM) tasks, since working memory is one of the domains primarily affected by mTBI. Notably, this ‘mild’ form of injury induces persisting cognitive dysfunction in approximately 15–20 percent of patients each year, exacting an enormous emotional and financial toll on society [18–21]. This work has led to mixed results, with some studies reporting hypoactivation in mTBI [22–24], others reporting hyperactivation [9, 17, 25, 26], and still others reporting both hypo- and hyper-activation [8, 27, 28]. This heterogeneity in results may be due to differences in the tasks that have been used in these studies (see [29] for a review). Another observation that is emerging from the mTBI literature is the importance of the integrity of the ‘default network’ (DN), a network of brain areas that is more active when subjects are “at rest” than when engaged in a cognitive task [30–32]. Importantly, activity in the DN appears to change as a function of recovery such that as post-concussive symptoms abate, metrics of functional connectivity return to normal levels [32]. The core regions of the DN include posterior cingulate cortex (PCC)/precuneus, inferior parietal lobule and medial prefrontal/perigenual anterior cingulate (ACC) [33].

While the work that has been conducted to date investigating the consequences of mTBI on brain activation has led to a better understanding of how the brain responds to trauma, only a few studies have studied subjects at time points within the first month following injury, and we are unaware of any studies to date that have enrolled patients at their initial medical contact in the emergency department setting. Moreover, few studies have followed their subjects longitudinally. Therefore, while the extant literature provides important insight about the reorganization of brain function, it is not generalizable to the wider context of acute head injuries, and cannot predict which subjects will recover and which will not.

Our objective was to understand the effect of concussive head injury on working memory, at the earliest possible time point after a concussion and in the first week of recovery. We compared patients diagnosed with mTBI in the emergency department to controls using the N-back task for working memory [8] and sequential neuroimaging at two early time points after trauma (0–3 days and 7–10 days post-injury). All subjects completed the least demanding condition of this task (the 0-back condition) at all time points. This task was used because it was sufficiently easy that all subjects were able to perform well, even immediately after sustaining an mTBI. Furthermore, low-load working memory tasks like the 0-back have been associated with hypoactivation in the mTBI literature [29], though this is not always the case (as noted above). Additionally, subjects completed additional, and more demanding, conditions of this task at the second time point (the 1-back and 2-back conditions). We hypothesized that clinical and fMRI measures of working memory would be abnormal in mTBI patients immediately after injury, but these measures would improve in those patients with clinical recovery within 1 week. Regarding the fMRI measures, we specifically predicted that the mTBI group would show hypoactivation on the 0-back task immediately after injury. We also hypothesized that increased memory workload at 1 week following injury would expose different activation patterns in those mTBI patients with persistent symptoms, compared to those with full clinical recovery.

## Methods

### Study Design and Setting

This was a prospective cohort study performed in a single tertiary care academic medical center with a level 1 trauma center and 60,000 annual emergency department patient visits. The University of Vermont’s Institutional Review Board (IRB) approved this study and written informed consent was obtained from all subjects prior to enrollment.

### Study Population

Research staff screened emergency department patients for the study’s inclusion and exclusion criteria. Eligible patients were those aged 18–60 years old who presented to the emergency department with isolated head injury, and medical diagnosis of a concussion based on standard emergency department criteria of Glasgow Coma Scale (GCS) > 14 at the time of injury. For purposes of the study, we defined an isolated head injury as head trauma with an injury severity score (ISS) for any other organ system <2 (i.e., “moderate” severity on a scale of 1 to 6, minor to maximal/untreatable). Concussion was defined as a patient with acute head trauma endorsing two or more of the following symptoms associated with their injury: loss of consciousness, persistent headache, blurred vision, confusion, dizziness, memory problems or poor balance. Head CT scans were performed at the discretion of the patient’s physician. Patients were excluded if: 1) they did not exhibit two or more concussive symptoms (above); 2) had a past history of a serious TBI (i.e., requiring surgical intervention); 3) they were unable to complete initial MRI within 72 hours of injury; 4) they had a pre-existing neurological disorder; 5) they had a psychiatric condition requiring medical treatment within the past year; 6) any contraindications to MRI scanning. Two control groups were also recruited: normal volunteers without acute injury who responded to flier advertisements and non-head injured patients who presented with an extremity injury to the emergency department within 72 hours of injury. Extremity injury was defined as a non-surgical injury to the arms or legs, including the shoulder and hips, with no head trauma.

### Clinical Data Collection and Processing

Initial assessments were completed by structured interviews performed by research staff, from review of emergency department charts, discussion with the patient’s physician, and evaluation of subjects while in the emergency department. Additional details on symptoms were also acquired from questionnaires and computer based assessments. Follow-up assessment was performed by a Research Associate at the time of repeat MRI and ImPACT testing Study data were collected and managed using REDCap electronic data capture tools hosted at UVM [34].

### fMRI task and stimuli

The N-back working memory task was used, at both Time 1 and Time 2; however, at Time 1 only the easiest condition (0-back) was used while at Time 2 three conditions were included (the 0-back, 1-back and 2-back conditions). All subjects were trained and checked for proficiency (correctly responding to all targets) by research staff prior to completing the tasks in the MRI scanner. The tasks were composed of series of letters that were presented on a projector screen, which the subject could see through a mirror mounted on the MRI’s head coil. Instructions for the tasks were as follows: 0-Back: “please respond when the letter you see matches the letter B (different letters were used at Time 1 and Time 2);” 1-Back: “please respond when the letter you see matches the letter you saw one letter back;” 2-Back: “please respond when the letter you see matches the letter you saw two letters back.” Subjects only responded to the letters when they met the above criteria and did so by pressing a button on a controller with their right index finger. When letters did not meet the above criteria subjects were asked not to respond. Each task lasted for approximately 3 minutes and a block design was used in which there were three blocks of the task (32 sec each) interleaved with blocks of rest (32 sec). The tasks were programed using E-Prime.

### MRI acquisition

Brain MRI data was acquired on a Philips Achieva 3.0 Tesla (Philips Healthcare, Best, Netherlands) scanner using an 8-channel head coil. T1-weighted images were acquired using a 3D inversion recovery spoiled gradient echo technique (TE/TR/TI/flip angle = 3.7ms/8.1ms/1008ms/8° with a SENSE factor of 1.5) to generate the Magnetization Prepared Rapid Acquisition Gradient Echo (MPRAGE) image. A sagittal acquisition matrix of 240x240x160 provided whole-brain coverage with an isotropic 1mm spatial resolution with a scan time of less than 8 minutes. The BOLD signal was captured with a T2* sequence (TE/TR/flip angle = 35ms/2000 ms/90°) with an in-plane resolution of 3 x 3 mm, and a slice thickness of 3.8 mm. For each of the n-back runs, 90 volumes were acquired (prior to the acquisition of these 90 volumes, an additional four were acquired (and discarded) to ensure steady-state magnetization).

### Outcome measures

Following the first MRI all subjects completed the Immediate Post-Concussion Assessment and Cognitive Testing (ImPACT) computerized neurocognitive testing battery. The ImPACT test measures attention span, working memory, sustained and selective attention, response variability, non-verbal problem solving and reaction time, each of which is sensitive for mild cognitive impairment. ImPACT also includes a symptom score based on patient self-report. All ImPACT testing was done in a small quiet conference room located just outside of the emergency department. Repeat MRI and ImPACT testing was done 7–10 days following injury. Post-concussive symptoms were obtained as part of the ImPACT test, and include 22 symptoms: headache, nausea, vomiting, balance problems, dizziness, fatigue, trouble falling, sleeping more than usual, drowsiness, sensitivity to light, sensitivity to noise, feeling dazed or stunned, irritability, sadness, nervousness, feeling more emotional than normal, numbness or tingling, feeling slowed down, feeling mentally foggy, difficulty concentrating, difficulty remembering, visual problems. Subjects rated each of these symptoms on a Likert scale ranging from 0 (the symptom was not experienced at all) to 6 (the symptom was the worst they had ever experienced). Among these symptoms, we defined cognitive symptoms as difficulty concentrating, difficulty remembering, feeling mentally foggy, feeling slowed down, drowsiness, fatigue and sleeping more than usual as previously defined by factor analysis [35]. The primary outcome of clinical recovery for individual patients, was defined as resolution of reported cognitive symptoms prior to time 2; other symptoms and ImPACT results were reported as secondary outcomes.

### Primary Data Analysis

All variables were summarized descriptively. The primary outcome of clinical recovery was assessed by determining the number of post-concussive symptoms at time 2. We report the mean number of symptoms for controls and mTBI subjects, with standard deviations, and the estimated magnitude of difference between groups with 95% confidence interval for this difference. We also report the mean and standard deviation of post-concussive symptoms between the subgroups of recovered and non-recovered subjects, along with the difference in means between these subgroups, with the 95% confidence interval. Secondary outcomes of neurocognitive testing results, were reported in the same fashion for both the experimental groups and subgroups. Statistical calculations were performed with SPSS (PASW Statistics 18, release 18.0.2).

### fMRI Data Analysis

All images were preprocessed using Analysis of Functional Neuro-Images (AFNI) [36]. The realignment, co-registration and normalization were done in a single transform. This was accomplished by (1) calculating and saving the parameters necessary for realignment (i.e., the spatial co-registration of all images in each time-series to the first image of the series). Next, (2) the parameters necessary to co-register the first image in each time-series with the high resolution MPRAGE were calculated and saved. Third, (3) the MPRAGE image was warped into standard space, and the warping parameters were saved. Finally, the transforms necessary to realign, co-register and warp the data into standard space were combined and applied to the functional time-series data in a single transformation. The images were then smoothed, using an 8 mm^3^ Gaussian smoothing kernel, and scaled to the mean intensity across time-points. The data were then deconvolved, using a boxcar function in which each condition was represented by a regressor (motion parameters and two polynomial regressors [to model signal drift] were included as regressors of no interest). The boxcar function was convolved with a gamma variate hemodynamic response function prior to fitting it in the model. Group-level statistics were performed using ANOVAs. All group-level statistical maps were thresholded using both the alpha level and cluster size (extent of activation). The alpha level was set at p<0.01 and the cluster size was set at 39 contiguous voxels in native space. The results of Monte Carlo simulations (using 3dClustSim) showed that this combination resulted in a corrected alpha level of p<0.05.

## Results

### Demographics

Participants consisted of 27 mild TBI patients, 10 orthopedic controls and 9 healthy controls (Table 1). Head CT was performed on 12 of the 30 mTBI subjects. One subject had a subtle, small area of intraparachymal hemorrhage. This subject was not excluded. She did not require surgery and recovered uneventfully. Of the entire sample, two mTBI patients and one healthy control did not return for their follow up scan and were therefore excluded from the sample. The mTBI patients were enrolled in the study within 72 hours of injury and while the research MRI scanner was available, and returned for a second scan 1 week later. No additional, focal lesions were identified upon qualitative radiological review of MR images in any of the subjects. Of the 25 mTBI patients eight mTBIs reported complete recovery from all cognitive symptoms between scan 1 and scan 2, ten mTBIs reported minimal recovery by the completion of the second scan, and the remainder (seven) reported moderate recovery. In the analyses below that compare recovered mTBIs to non-recovered mTBIs, only the eight mTBIs who had symptoms at Time1 and who recovered fully by Time 2 and the 10 mTBIs who reported minimal recovery by Time 2 were included. By including only the two ‘tails’ of the distribution of recovery in this way (the recovered and non-recovered), we sought to maximize the difference associated with recovery. There were no differences between groups on any of the demographic variables (see Table 2 for additional demographic information).

**Table 1:** Demographics of the groups.

Demographics						
	Healthy Controls	All mTBI		mTBI Cog. Rec.	mTBI No Cog. Rec.	
N	18	25		8	10	
Gender	44% Male	44% Male		38% Male	60% Male	
Handedness	83% Right	88% Right		75% Right	100% Right	
Loss of Consciousness	0%	28%		30%	13%	
Abnormal CT	0%	4%		13%	0%	
	*Mean (SD)*	*Mean (SD)*	*Diff*. *(95% CI)*	*Mean (SD)*	*Mean (SD)*	*Diff*. *(95% CI)*
Age (years)	28.0 (9.2)	27.8 (11.1)	0.28 (-6.0,6.6)	30.0 (15.6)	26.2 (9.6)	3.8 (-10.0,17.6)
Education (years)	15.7 (2.4)	14.7 (2.3)	0.9 (-0.6,2.4)	13.7 (1.9)	15.1 (2.0)	1.4 (-0.6,3.4)
Time1 (days since injury)	2.2 (0.8)	2.0 (0.9)	0.3 (-0.4,1.0)	2.2 (1.0)	2.0 (0.6)	-0.2 (-1.0,0.6)
Time2 (days since injury)	9.5 (1.6)	8.7 (1.2)	0.7 (-0.5,1.9)	9.0 (1.3)	9.0 (1.2)	0.1 (-1.2,1.4)
Time1 to Time2 (days)	7.4 (1.5)	6.7 (1.0)	0.6 (-0.3,1.4)	6.7 (1.2)	7.0 (1.2)	0.3 (-0.59,1.5)
initial GCS	15 (0)	15 (0)	n/a	15 (0)	15 (0)	n/a

**Table 2:** Additional demographic information.

Setting of Injury						
	Motor vehicle crash	Sports	Fall from standing	Fall from height	Assault	Other
All mTBI	4%	52%	24%	4%	8%	8%
mTBI Cog. Rec.	0%	63%	37%	0%	0%	0%
mTBI No Cog. Rec.	10%	20%	20%	10%	20%	20%

### Clinical Outcomes and Predictive Power of Clinical Measures

The number of post-concussive symptoms at time 2 was significantly higher in mTBI subjects compared to controls as expected. In comparison of subjects with cognitive recovery to those with no cognitive recovery (primary outcome), there were no differences in demographics, initial symptoms or neurocognitive measurements (verbal memory, visual memory, visuo-motor speed, reaction time [RT], impulse control, and cognitive efficiency index) at the initial time point. Because only one subject had an abnormal CT scan, and structural MRI did not reveal additional lesions, none of these clinical measures provided additional predictive power in determining the primary outcome. The secondary outcomes of neurocognitive results at time 2 were similar at both time points for those subjects with and without resolution of cognitive symptoms as shown by the overlapping 95% confidence intervals for each of these measures (see Table 3).

**Table 3:** Clinical outcomes. Symptom and neurocognitive testing scores for control, mTBI and cognitive recovery subject subgroups.

	Controls	mTBIs	Difference Between Experimental Groups	Cognitive Recovery*	No Cognitive Recovery	Difference Between Subgroups
N	10	25		8	10	
Total Symptom Scores **	*Mean (SD)*	*Mean (SD)*	*Difference (95% CI)*	*Mean (SD)*	*Mean (SD)*	*Difference (95% CI)*
# Symptoms (T1)†	3.4 (5.4)	27.4 (18.2)		22.1 (20.5)	34.6 (13.2)	-12.5 (-31.0,6.1)
# Symptoms (T2) †	2.1 (3.2)	13.1 (15.2)		4.0 (4.5)	25.3 (17.9)	-21.3 (-34.3,-8.3)
# Cog Symptoms (T1) †	0.6 (1.1)	13.8 (9.1)		11.5 (9.9)	18.3 (7.4)	-6.8 (-16.1,2.4)
# Cog Symptoms (T2) †	1.4 (2.6)	7.0 (8.8)		0 (0)	15.1 (8.8)	-15.1 (-21.4,-8.8)
***Neurocognitive Testing Score***	*Mean (SD)*	*Mean (SD)*	*Difference (95% CI)*	*Mean (SD)*	*Mean (SD)*	*Difference (95% CI)*
Verbal Memory (T1)§	80.8 (12.5)	82.3 (11.7)	-1.5 (-11.3,8.4)	77.7 (9.0)	85.9 (10.8)	-8.2 (-18.0,3.23)
Visual Memory (T1) §	68.9 (14.0)	66.2 (18.7)	2.7 (-9.5,14.9)	57.2 (13.2)	64.1 (22.1)	-6.8 (-26.0,12.3)
Visual Motor Speed (T1) §	37.9 (8.9)	40.0 (9.8)	-2.1 (-9.4,5.1)	36.2 (11.8)	38.9 (9.9)	-2.7 (-14.8,9.4)
Reaction Time (T1) §	0.58 (0.11)	0.68 (0.15)	-0.07 (-0.16,0.31)	0.70 (0.2)	0.65 (0.15)	0.04 (-0.15,0.25)
Impulse Control (T1) §	6.4 (5.2)	4.3 (2.6)	2.1 (-1.9,6.2)	5.3 (3.7)	3.9 (2.0)	1.4 (-2.1,4.9)
Cognitive Efficiency Index (T1) §	0.23 (0.20)	0.27 (0.21)	-0.04 (-0.20,0.12)	0.26 (0.15)	0.31 (0.25)	-0.054 (-0.27,0.16)
Verbal Memory (T2) §	82.5 (16.3)	90.4 (9.1)	-7.9 (-19.9,4.1)	90.1 (9.5)	87.2 (10.0)	2.9 (-6.9,12.7)
Visual Memory (T2) §	71.3 (15.7)	70.7 (14.0)	0.58 (-11.5,12.7)	68.6 (13.0)	67.0 (17.0)	1.63 (-13.3,16.6)
Visual Motor Speed (T2) §	40.6 (9.0)	41.5 (9.5)	-0.87 (-8.1,6.3)	39.0 (10.9)	40.1 (10.4)	-1.1 (-11.9,9.7)
Reaction Time (T2) §	0.56 (0.15)	0.60 (0.14)	-0.03 (-0.15,0.08)	0.59 (0.10)	0.64 (0.20)	-0.055 (-0.21,0.10)
Impulse Control (T2) §	5.6 (4.6)	4.8 (3.1)	0.76 (-2.9,4.4)	5.4 (3.7)	5.1 (3.4)	0.28 (-3.4,3.9)
Cognitive Efficiency Index (T2) §	0.36 (0.21)	0.39 (0.19)	-0.02 (-0.19,0.14)	0.40 (0.18)	0.35 (0.23)	0.054 (-0.16,0.26)

N.B., At baseline recovered subjects did not differ from non-recovered subjects in symptom or neurocognitive scores

* Cognitive recovery was defined as resolution of cognitive symptoms (difficulty concentrating, difficulty remembering, feeling mentally foggy, feeling slowed down, drowsiness, fatigue and sleeping more than usual)

** Total of 22 post-concussive symptoms, including those defined as cognitive symptoms

† Significant at p<0.001 between Controls and mTBIs

§ no significance between recovered and non-recovered.

### Extremity Injured controls vs. Healthy Controls (HC)

We first compared the two groups of Controls to one another to ensure that there were no differences between these two Control groups. This was done by comparing the 0-back data at Time1 and Time2 (in separate t-tests). No differences were found in either behavioral accuracy or in the imaging data. The RTs for extremity injured controls were faster on the 0-back task at both time points (p < 0.04 and p < 0.03 for Time1 and Time2 respectively); however, because this difference suggested that the information processing of the extremity injured controls was (if anything) more efficient than the other HCs (rather than the reverse), and because their accuracy was comparable, and because their RTs were not different on the 1-back and 2-back tasks, we felt that this difference was likely due merely to random chance rather than to a systematic difference between the groups. Therefore in all subsequent analyses, these two groups of Controls were pooled to form a single Healthy Control (HC) group.

### Behavior

For both response time (RT) and accuracy (d’), we conducted two ANOVAs. Because the accuracy data was not normally distributed, it was first log-transformed to assure normality. The first compared 0-back performance across time. This was a 2 X 3, mixed between- and within-subjects ANOVA with time as the within-subjects factor (Time 1 vs. Time 2) and group as the between-subjects factor (HC, mTBIrec, mTBInonrec). For both RT and d’, none of the main effects or interactions were significant (see Table 3).

The next analysis interrogated task difficulty by comparing the three levels of n-back working memory load across the three groups at Time2 (see Table 4). This was a 3 x 3 mixed between- and within- subjects ANOVA, with load as the within-subjects factor (0-back, 1-back, 2-back) and group as the between-subjects factor (as before). For both RT and d’, a Greenhouse-Geisser correction for non-sphericity was used (based on Mauchly’s test of sphericity). In the RT data, there was a main effect of load (F(1.57,50.14) = 8.89, p < 0.001). Paired t-tests showed that this main effect resulted from subjects responding with longer latencies on the 2-back than on the 1-back (t(34) = 3.59, p < 0.001), while the difference between 0-back and 1-back was not reliably different. For d’, the overall pattern of results was the same: a main effect of working memory load (F(1.33, 41.21) = 12.76, p < 0.0001) and no other effects or interactions. Furthermore, as in the RT data, the effect of load was due to subjects being less accurate on the 2-back than on the 1-back (t(34) = 4.13, p < 0.0001), while the difference between 0-back and 1-back was not significantly different.

**Table 4:** Behavioral performance on the N-Back task performed in the scanner (mean, S.D.).

Behavior				
	Time1	Time2		
*Response Time*	0back	0back	1back	2back
Healthy Controls	583.58 (104.16)	618.23 (107.38)	630.84 (106.80)	699.24 (145.87)
mTBIrec	653.61 (74.01)	661.87 (73.96)	654.35 (74.17)	712.75 (79.89)
mTBInonrec	624.94 (103.84)	654.88 (159.99)	660.34 (143.61)	707.70 (235.64)
*Accuracy (d’)*				
Healthy Controls	3.77 (0.26)	3.52 (0.45)	3.68 (0.35)	2.92 (0.85)
mTBIrec	3.61 (0.51)	3.27 (0.73)	3.78 (0.21)	2.88 (0.68)
mTBInonrec	3.47 (0.68)	3.74 (0.25)	3.49 (0.52)	2.60 (0.82)

### MRI Data

#### mTBI vs. Healthy Controls, change over time

**Fig 1:**
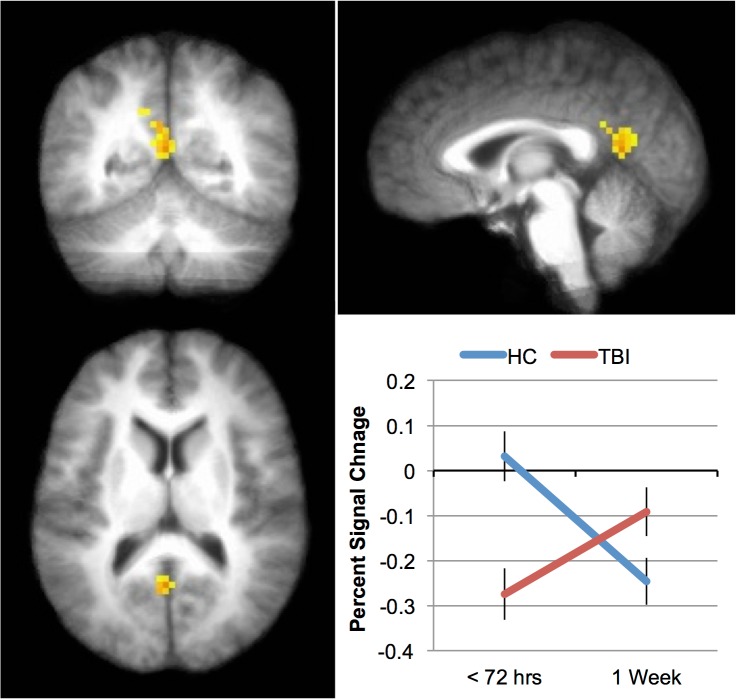
Differences in activation patterns between mTBI and healthy controls over time. The interaction between Group (TBI vs. HC) and Time (Time 1 vs. Time 2) during 0-back task was significant in the posterior cingulate cortex (PCC). The inset graph shows the percent signal change. Error bars represent the standard error of the mean (SEM).

This 2 X 2 ANOVA, with factors of group (mTBI vs. HC) and time (Time 1 vs. Time 2), was conducted using 3dLME.R, a script distributed with the AFNI suite of image analysis tools that implements the R statistical analysis software. Only the 0-back data was included since only these data were collected at both time points. There was a main effect of Time in several areas including superior frontal gyrus, inferior frontal gyrus, insula and inferior parietal lobule (see Table 5). In all cases, this was because the activation was greater at Time 1 than at Time 2. The main effect of Group was not reliable in any brain area. However, there was an interaction between Group and Time in the posterior cingulate cortex (see Table 5 and Fig 1).

**Table 5:** Brain regions showing reliable activation in the analysis of mTBI vs. HCs (B.A. = Brodmann’s area).

mTBI vs. Healthy Controls, change over time					
	*BA*	X	Y	Z	F-value*
*Time (Time 1 vs*. *2)*					
Superior Frontal Gyrus	*10*	25	46	23	18.19
Superior Frontal Gyrus	*9*	-25	37	32	14.98
Inferior Frontal Gyrus	*45*	-52	19	8	14.14
Insula	*13*	-28	13	-6	17.95
Inferior Parietal Lobule	*40*	49	-52	38	15.90
*Group (mTBI vs*. *HC) X Time (Time 1 vs*. *2)*					
Posterior Cingulate	*23*	-1	-55	14	11.78

* the F-value is for the voxel of maximal intensity in each cluster.

#### Recovery vs. non-Recovery

**Fig 2:**
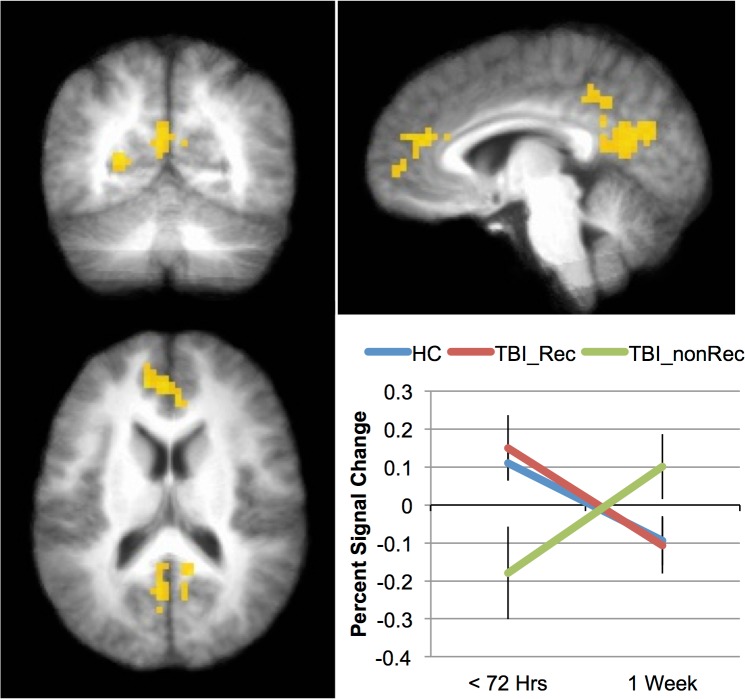
Indices of recovery over the first week post-injury. Areas showing an interaction between Group (TBI_Rec, TBI_nonRec, HC) and Time (Time 1 vs. Time2) included posterior cingulate and medial frontal cortices (yellow). The inset graph shows the interaction in the medial frontal region. Error bars represent standard error of the mean (SEM).

In the analyses that specifically investigated recovery, we compared those who made a good recovery (8 mTBIs) to those who made minimal or no recovery (10 mTBIs). This 3 X 2 ANOVA, with factors of group (mTBI-Rec, mTBI-nonRec, HC) and time (Time 1 vs. Time 2) was similar to the analysis above. As in the previous analysis, only the 0-Back data was analyzed. There was a main effect of Group in both the paracentral lobule and precentral gyrus (see Table 6), and in both cases this was due to more activation in the HC and mTBI-Rec than the mTBI-nonRec (the HC and mTBI-Rec groups did not differ). There was also a main effect of Time in superior and inferior frontal areas, the insula and in inferior parietal areas (see Table 6). In all cases, this was because subjects showed more activation at Time 1 than at Time 2. Finally, as Table 6 and Fig 2 show, there was an interaction between Group and Time in four regions: medial prefrontal, posterior cingulate, parahippocampal gyrus and precuneus. The activation across the three groups for the medial prefrontal area is shown in Fig 2: while the HCs and the mTBI-Rec show a comparable pattern of activation, the activation seen in the mTBI-nonRec over time is markedly different (the pattern of activation seen for the other areas was substantially the same).

**Table 6:** Brain regions showing reliable activation in the analysis of mTBI-Rec vs. mTBI-nonRec vs. HCs. (B.A. = Brodmann’s area).

Recovery vs. non-Recovery					
	*BA*	X	Y	Z	F-value*
*Group (mTBI-Rec*, *mTBI-nonRec*, *HC)*					
Paracentral Lobule	*31*	-1	-28	44	9.61
Postcentral Gyrus	*3*	58	-22	41	7.96
*Time (Time 1 vs*. *2)*					
Superior Frontal Gyrus	*10*	25	43	20	16.59
Inferior Frontal Gyrus	*45*	-52	19	8	14.75
Insula	*13*	-28	13	-6	21.51
Inferior Parietal Lobule	*40*	49	-52	38	19.81
*Group (mTBI-Rec*, *mTBI-nonRec*, *HC) X Time*					
Medial Frontal Gyrus	*9*	-1	43	20	10.57
Posterior Cingulate	*23*	-4	-58	17	8.46
Precuneus	*7*	-10	-46	44	10.40
Parahippocampal Gyrus	*18*	-22	-55	8	7.74

* the F-value is for the voxel of maximal intensity in each cluster.

#### mTBI vs. Healthy Control, final follow-up

**Fig 3:**
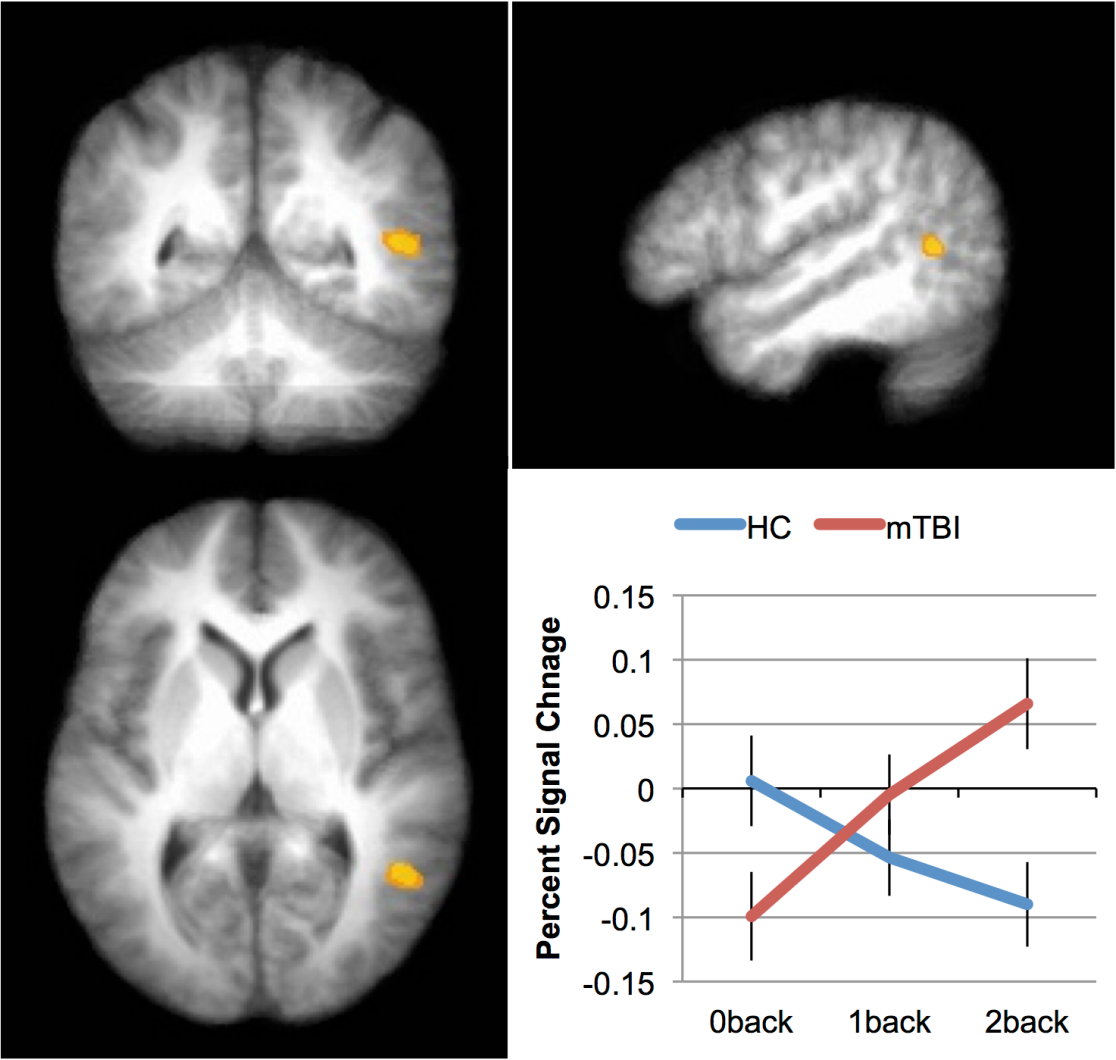
Differences in activation patterns between patients and controls in response to increased working memory load. The area showing an interaction between Group (mTBI vs. HC) and Load (0back, 1back, 2back) at Time 2 (the middle temporal gyrus). The inset graph shows this interaction. The error bars represent the standard error of the mean (SEM).

We investigated the differential effect of task difficulty (working memory load) across the two Groups (mTBI vs. HC) with a 2 X 3 ANOVA. The factors were group (mTBI vs. HC) and load (0-, 1-, 2-back). Because all three loads were used only at Time 2, only data from this time point was included in this analysis. Of the main effects, only the effect of Load was reliable (see Table 7). The main effect of Load was seen in the expected fronto-parietal network, including superior and middle frontal areas, parietal areas (precuneus), as well as basal ganglia (caudate) and cerebellar regions. While there was no main effect of Group, a significant group difference was found for the 2-back, with the only area to show an interaction between group and load was in the middle temporal gyrus (see Table 7 and Fig 3). As can be seen in the plot in Fig 3, the interaction resulted from individuals with mTBI increasing the activation in this area across the three levels of load; the HCs, in contrast, decreased the activation in this area as the N-Back task became more difficult. This may be due to differential strategies employed by the two groups, with mTBIs increasingly relying on long-term memory structures while HCs rely more heavily on other (likely frontal) structures.

**Table 7:** Brain regions showing an interaction between Group and Load at 1 week post injury.

mTBI vs. HC, final follow-up					
	*BA*	X	Y	Z	F-value*
*Load (0-*, *1-*, *2-Back)*					
Superior Frontal Gyrus	*9*	-22	43	14	15.05
Middle Frontal Gyrus	*6*	-22	-10	50	35.35
Middle Frontal Gyrus	*6*	25	-10	50	41.54
Inferior/Middle Frontal Gyrus	*47*	-22	34	-9	12.41
Insula	*13*	-25	22	8	19.38
Caudate Head	*-*	16	16	2	20.08
Precuneus	*7*	7	-61	44	24.97
Parahippocampal Gyrus	*36*	-37	-25	-12	19.36
Culmen	*-*	-4	-34	-15	15.22
Cerebellar Tonsil	*-*	40	49	-30	16.61
*Group (mTBI vs*. *HC) X Load (0-*, *1-*, *2-Back)*					
Middle Temporal Gyrus	*39*	50	-52	8	8.37

* the F-value is for the voxel of maximal intensity in each cluster.

#### Group X Load analysis as a function of recovery

**Fig 4:**
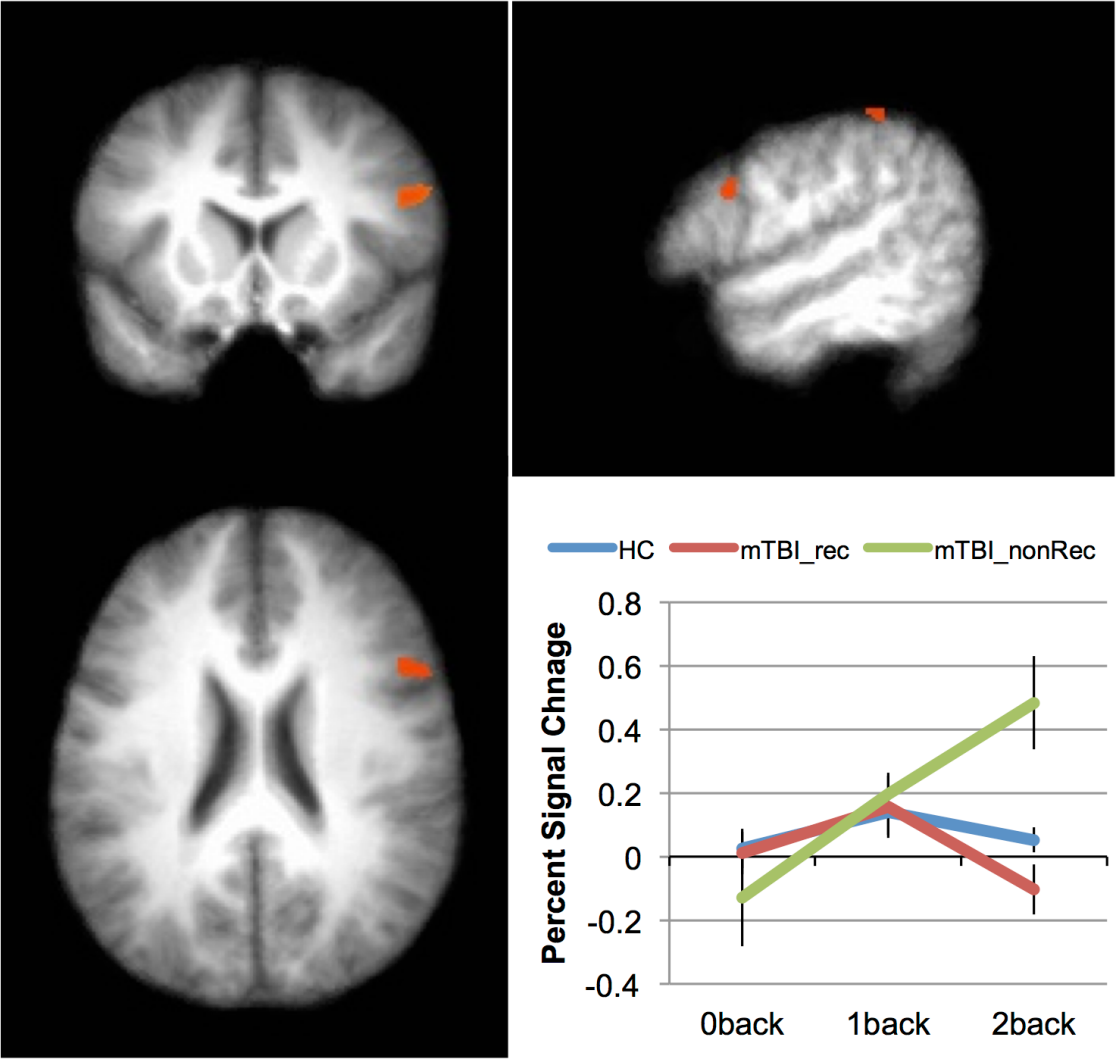
Indices of recovery as a function of increased memory load. Areas showing an interaction between Group (mTBI-Rec, mTBI-nonRec, HC) and Load (0back, 1back, 2back). The inset graph shows the interaction for the inferior frontal region. Error bars represent the standard error of the mean (SEM).

The above analysis was rerun, but with the mTBI group divided into those who showed good recovery vs. those who did not. The factors were the same, but the factor of group had three levels (mTBI-Rec, mTBI-nonRec, HC) rather than two. As in the above analysis, there were no areas that showed a main effect of group. Furthermore, the effect of Load was found in largely the same set of areas as in the above analysis (see Table 8). No areas showed an interaction. However, when the cluster-level threshold was relaxed from 39 contiguous voxels to 30, two areas emerged: inferior frontal gyrus, and inferior parietal lobe. These can be seen in Fig 4 and Table 8.

**Table 8:** Brain regions showing an interaction between Group and Load at 1 week post injury.

Recovery vs. non-Recovery, final follow-up					
	*BA*	X	Y	Z	F-value*
*Load (0-*, *1-*, *2-Back)*					
Middle Frontal Gyrus	*6*	25	-10	50	31.54
Insula	*13*	-25	22	8	16.05
Caudate Head	*-*	16	16	2	17.78
Parahippocampal Gyrus	*36*	-37	-25	-12	15.5
Culmen	*-*	-4	-34	-15	14.47
Cerebellar Tonsil	*-*	40	-49	-30	15.53
*Group (mTBI-Rec*, *mTBI-nonRec*, *HC) X Load*					
Inferior Parietal Lobe	*40*	64	-37	32	4.63
Inferior Frontal Gyrus	*9*	49	13	23	5.07

* the F-value is for the voxel of maximal intensity in each cluster.

## Discussion

In this study, we set out to better understand the consequences of mild TBI on brain function, focusing on the first week after injury. We scanned individuals with mTBI twice: first within 72 hours of their injury to capture acute changes, and then 7–10 days later to assess recovery. This is the first study to our knowledge to longitudinally investigate working memory within the first week on injury in mTBI patients. We also differentiated between those patients who reported cognitive recovery, and those with persistent cognitive symptoms. Demographics, initial symptoms, neurocognitive measures at time 1, CT scans, and structural MRI, did not provide any predictive power to discriminate the primary outcome of recovery at 1 week. The failure of standard clinical assessment to identify those individuals at risk for post-concussive syndrome highlights the urgency of the current work.

While there is a growing body of work investigating working memory in TBI [5–17], much of this work has examined chronic injury, with scans acquired months—often years—after the injury has occurred. Much can be learned from this work (e.g., the reorganization of the nodes in the working memory network following insult); however, it is unclear from this work whether the reorganization that is seen in chronic samples is qualitatively similar to acute changes in brain function. The results of the current study show a pattern of results that is qualitatively similar to this previous work in individuals who do not recover at 1 week after injury (Fig 4): increased activation in the right [5, 13], often prefrontal [6], cortex when the activation of individuals who have sustained a TBI is compared with controls. However, this pattern is not evident in individuals who do recover. Thus, these data both replicate and extend previous findings.

While the replication of previous results in an acute sample is encouraging, the data from the 0-back task collected within 72 hours of injury is perhaps more important (Fig 2). These data show that areas of the ‘default network’ (DN) (posterior cingulate cortex (PCC) and medial prefrontal cortex (MPFC)) appear to predict outcome in this sample. The DN is a set of regions that have been shown to be significantly more active when subjects are at rest (or engaged in a task with low cognitive load) than when they are engaged in effortful processing (e.g., [33, 37], and while the DN is comprised of more brain regions than the PCC and MPFC, these two regions appear to be most reliably associated with it. In the TBI literature, DN activity has been shown to predict recovery by Han et al. [32] in US Military personnel when the initial scan was acquired approximately 30 days after the injury rather than within 72 hours, as here. Therefore, the current work suggests that the changes evident in the DN occur shortly after the injury, and that the pattern of changes have the potential to be predictive of recovery. When the activation in these areas was low during the 0-back task (hypoactivation), individuals with a mild TBI did not recover; when activation in these areas was relatively high during the 0-back task, individuals with mTBI did recover. This result raises the possibility that the activation in the DN may eventually be useful to identify individuals who will require more intensive rehabilitation after mTBI.

While any interpretation of these findings must remain highly speculative, it is possible that hypoactivation of the DN is a signal that the brain has to work harder to accomplish even the relatively simple 0-back task. This interpretation is suggested by work such as Singh and Fawcett [38], who showed that the DN deactivates as a function of task load: as a task becomes more difficult, subjects deactivate their DN. It is therefore tempting to conclude that the mTBIs in our sample who showed hypoactivation in their DNs and did not recover had to expend more cognitive resources on the 0-back task than the subjects who were able to maintain activation in their DNs and later recovered. Why this would be so is not possible to know in the current sample, but is likely to be a fruitful avenue of future work.

One seemingly surprising result from the comparison of the 0-back data across time (72 hours after injury vs. 1 week after injury) is that the activation appears to decrease in the DN across time in the HCs and mTBIs who recovered. Given that one might expect the task to become ‘easier’ with practice, one would expect the DN activation to increase. This is because prior research has shown that DN activity decreases as tasks become more difficult (and, conversely, increases as tasks become easier) (e.g., [38]). However, the comparison of the 0-back task at the two time-points in the current study is complicated by the fact that at Time 2 (1 week after injury) all three n-back tasks were administered in randomized order. Thus, the 0-back task at Time 2 was qualitatively different from the 0-back task at Time 1 because of the effect of context. At Time 2, subjects had had experience of 1-back and 2-back as well as 0-back, and therefore the responses appropriate for the other n-back tasks (1-back and 2-back) had to be inhibited during the performance of the 0-back task. This change in task context may explain why the activation in the DN was less at Time 2 than at Time 1 for the HCs and for the mTBIs who recovered. This hypothesis does not explain why activation increased in the DN for the mTBIs who did not recover, and further work is required to fully understand this.

One of the strengths of our study is a highly characterized TBI population. We enrolled subjects in the emergency department and performed structured interviews and neurocognitive testing at the bedside. At initial presentation after head injury, our research staff was able to ascertain reliable information about the event, which is subject to recall bias in other studies that have enrolled patients at later time points. We performed comprehensive clinical evaluations at follow-up to provide characterization of functional recovery in the early phase after mTBI (a comprehensive clinical evaluation was also performed at initial presentation). A second strength is the early neuroimaging obtained by bringing emergency department patients directly to a research magnet. This study design allowed us to obtain a large series of MRIs with corresponding clinical information during the early (<1 week) time period after mTBI. It is only through obtaining data at multiple time points that the mechanisms of functional recovery can be inferred.

Our exclusion criteria might be considered less stringent than other studies, because we enrolled all adult emergency department patients with mTBI, only excluding past history of TBI requiring surgical intervention, psychiatric condition within the last year, or active alcohol/ drug intoxication. The less stringent exclusion criteria might also be considered a strength of our study because it makes our results more generalizable to the larger population of emergency department patients with heads injures.

Despite our attempts to include a diverse population of mTBI patients, our sample may not represent the whole mild traumatic brain injury population. We did not include children or older adults (our cutoff was 60 years of age). Individuals with alcohol and/or drug intoxication also make up a significant portion of emergency medicine trauma cases, but were not represented in this study.

Additionally, we were unable to capture the “true” initial time point after injury, and the timing of the first MRI varied between 11 hours and 72 hours after trauma. Functional recovery after brain injury may begin to occur immediately, and the earliest changes in cortical activation patterns would not be detected with our study design. The injury mechanisms were variable and although we documented the patient’s report of the mechanism, the actual injury parameters (such as impact angle and force, which can be measured through linear accelerometers in a cap or helmet) are unknown. Another limitation is that the outcome measure of clinical recovery was based on patient report. This may be problematic, because patients may have deficits that are not apparent to them, but only manifested on neurocognitive testing. In fact, our secondary outcomes of neurocognitive testing did not reveal significant differences in cognitive performance in patients who reported cognitive recovery compared to those who did not. Without pre-injury neurocognitive testing the results of tests obtained after injury can only be compared to standardized databases and tracked over time. However, inasmuch as pre-injury testing was not available on the individuals in this study, we felt that the patient’s report of cognitive improvement and resolution of symptoms was the most valid clinical outcome measure.

While the findings reported here are encouraging, they are just the first step towards predicting recovery in mTBI. Few clinics have the facilities to scan every patient who presents with a mTBI, and even fewer have the resources to do so. Future work will be to develop inexpensive, sensitive, diagnostic measures that correlate with changes in brain activation, and that can be easily administered in clinical settings to predict recovery.

## Conclusions

We describe patterns of brain activation during working memory tasks in the acute and sub-acute phases after mTBI. Normalization of activation patterns in the posterior cingulate cortex, a major node of the default network [33], corresponded to clinical recovery. Additionally, stronger engagement of working memory resulted in increased, possibly compensatory, recruitment of right-lateralized frontal and parietal regions in patients who did not recover 1 week after the injury. Our results provide critical insight into functional activation of working memory networks during the early phase of recovery after mTBI.
